# Well-Being Is Associated With Local to Remote Cortical Connectivity

**DOI:** 10.3389/fnbeh.2022.737121

**Published:** 2022-03-11

**Authors:** Yubin Li, Chunlin Li, Lili Jiang

**Affiliations:** ^1^CAS Key Laboratory of Behavioral Science, Institute of Psychology, Beijing, China; ^2^Department of Psychology, University of Chinese Academy of Sciences, Beijing, China

**Keywords:** MRI, well-being, ReHo, functional connectivity, local-to-remote cortical connectivity

## Abstract

Wellbeing refers to cognitive and emotional appraisal of an individual’s life and social functioning, which is of great significance to the quality of life of an individual and society. Previous studies have revealed the neural basis of wellbeing, which mostly focused on human brain morphology or network-level connectivity. However, local-to-remote cortical connectivity, which plays a crucial role in defining the human brain architecture, has not been investigated in wellbeing. To examine whether wellbeing was associated with local-to-remote cortical connectivity, we acquired resting-state images from 60 healthy participants and employed the Mental Health Continuum Short Form to measure wellbeing, including three dimensions, namely, emotional wellbeing, psychological wellbeing, and social wellbeing. Functional homogeneity (ReHo) and seed-based functional connectivity were used to evaluate local-to-remote cortical connectivity in these participants. For local connectivity, our results showed that ReHo in the right orbitofrontal sulcus was significantly positively correlated with psychological wellbeing but negatively correlated with social wellbeing. For remote connectivity, connectivity within the right orbitofrontal cortex and interhemispheric connectivity of the orbitofrontal sulcus were both positively associated with psychological wellbeing; functional connectivity between the right orbitofrontal sulcus and the left postcentral sulcus was positively associated with social wellbeing. Our results showed that wellbeing was indeed associated with local-to-remote cortical connectivity, and our findings supplied a new perspective of distance-related neural mechanisms of wellbeing.

## Introduction

Over decades, the increasing attention of neuroscientists has been devoted to wellbeing, which is a complex construct that pursuits optimal experience and functioning (Ryan and Deci, 2001). Wellbeing could be divided into two distinct aspects: one is hedonic wellbeing, as the hedonic aspect of subjective wellbeing, which means pleasure or happiness attainment (Diener, 1994), and the second is eudaimonic wellbeing, which means the actualization of human potentials or true value (Waterman, 1993; Ryan and Deci, 2001). Recently, Keyes (2009) integrated previous definitions of happiness and diagnosis methods of mental health and proposed that positive mental health conditions can be evaluated on the following three aspects, namely, emotional wellbeing, psychological wellbeing, and social wellbeing. Such a concept of the mental health continuum could also be used in neuropsychiatric patients (Keyes, 2007). Wellbeing is so important for the quality of an individual’s life and society, and studies on wellbeing are also important to behavioral sciences (Diener et al., 2003). In a summary, exploring the neural mechanisms of wellbeing could enrich our comprehension of how to live a happy life.

Recently, with the development of brain imaging techniques, an increasing number of neuroscientists began to investigate the neural mechanisms of wellbeing. Most of them used structural MRI (sMRI) or resting-state functional MRI (r-fMRI) to measure the human brain and used social wellbeing and subjective wellbeing to measure wellbeing. For the brain structure, Kong et al. (2015a,2019) employed the voxel-based morphometry method to reveal that both gray matter density in the orbitofrontal cortex (OFC) and gray matter volume in the left mid-dorsolateral prefrontal cortex (mid-DLPFC) were negatively correlated with social wellbeing. Meanwhile, eudaimonic wellbeing was associated with insular cortex volume (Lewis et al., 2014). Subjective wellbeing was reported to be related to subcortical brain volumes (Van’t Ent et al., 2017) as well as brain morphology in the superior temporal gyrus and the insula (Song et al., 2019). For brain function, using the regional fractional amplitude of low-frequency fluctuation (fALFF) method, Kong et al. (2016a) demonstrated that social wellbeing was positively associated with the fALFF method in the bilateral posterior superior temporal gyrus, right anterior cingulate, right thalamus, and right insula. Subjective wellbeing was negatively correlated with the fALFF in the bilateral superior frontal gyrus (SFG) and the right OFC (Kong et al., 2015b), together with that, eudaimonic wellbeing was positively correlated with fALFF in the right posterior superior temporal gyrus and the thalamus and negatively correlated with the strength of thalamic-insular connectivity (Kong et al., 2015c). There were also studies reporting that wellbeing was associated with within-network connectivity rather than between-network connectivity (Heller et al., 2013; Luo et al., 2017; Shi et al., 2018; Ren et al., 2019; Li et al., 2020), and the main networks included the fronto-parietal network, the default-mode network, and the insular network. Kong et al. (2016b) also demonstrated that local functional connectivity ReHo in the inferior frontal gyrus and the OFC was associated with wellbeing. One note was that many of the above studies detected significant mediation effects such as personality, emotional intelligence, mindfulness, pursuit, and thought patterns associated with brain-wellbeing (Vatansever et al., 2020). Since wellbeing was such a general construct involving a lot of psychological processes such as cognitive, emotional, and self, the first step should be clarifying its neural basis but not involve another behavioral measurement.

Most previous studies on wellbeing focused on correlations between wellbeing and gray matter volume, gray matter density, or r-fMRI metrics, such as fALFF and ReHo, which mainly concentrated on local characteristics of the human brain. There were few studies combining local short-range functional connectivity and remote long-range functional connectivity. Anatomical distance is of great significance to human brain architecture regarding the cost and efficiency of information processing (Jiang et al., 2015). A previous study has revealed that the development of large-scale brain networks was characterized by the weakening of short-range functional connectivity and the strengthening of long-range functional connectivity (Supekar et al., 2009). Also, individual connectivity variability related to the cortical hierarchy positively correlated with the degree of long-range connectivity but negatively correlated with the local connectivity (Mueller et al., 2013). In addition, the anatomical distance of functional connectivity played important roles on patient brains of various diseases. For example, one study suggested that the long-range connections linking dorsal anterior cingulate to posterior cingulate and precuneus should be considered as a candidate locus of dysfunction in attention-deficit hyperactivity disorder (ADHD; Castellanos et al., 2008). Guo et al. (2015) demonstrated a pattern of increased long-range connectivity in the posterior default-mode network (DMN) in schizophrenia. From the perspective of information transferring and dynamics, long-range functional connectivity was based on short-range functional connectivity as well as neurodevelopment through short-range functional connectivity (Jiang et al., 2015). Therefore, it was important to investigate local-to-remote cortical connectivity in wellbeing for a complete picture of brain mechanisms of wellbeing.

In this study, we employed a novel seed-based method, which mainly included the computation of 2dReHo (Zuo et al., 2013) and seed-based functional connectivity (SFC), to examine local-to-remote cortical connectivity (Jiang et al., 2015) in individual wellbeing. We aimed to investigate whether wellbeing correlated with the brain spontaneous activity as well as the relations between wellbeing and local-to-remote functional connectivity. Studies on neural mechanisms of wellbeing not only enriched our comprehension of wellbeing and how to live a happy life but studies on the local-to-remote cortical connectivity might also shed new insights on wellbeing studies from perspectives of both neuroimaging and behavior.

## Materials and Methods

### Participants

A total of 67 healthy subjects (32 men, age 18.6–64.3 years) were recruited from the local community or universities by advertisements. All the participants underwent a detailed mental health interview by two trained psychologists using the Mini-International Neuro-Psychiatric Interview. People with a history of major neuropsychiatric illness, head injury, alcohol, and drug abuse were excluded. They were also assessed with the Wechsler Adult Intelligence Scale-4th Edition (in Chinese, WAIS-IV), the Schutte Self-Report Emotional Intelligence scale in Chinese Version (SSEIS), State-Trait Anxiety Inventory, Mental Health Continuum-Short Form (MHC-SF), Emotion Regulation Questionnaire, Chinese Perceived Stress Scale, the Achievement Motivation Scale, and the Self-Control Scale. The Institutional Review Board of the Institute of Psychology, Chinese Academy of Sciences approved this study, and written informed consent was obtained from individual participants prior to data acquisition.

### Behavior Measurement

We employed a 14-item version of MHC-SF to measure the wellbeing of the participants, which was composed of emotional wellbeing, psychological wellbeing, and social wellbeing (Keyes, 2007). Specifically, emotional wellbeing reflects the hedonic aspect of wellbeing that encompasses positive affective states and high levels of life satisfaction (Diener et al., 2003). Psychological and social wellbeing are collectively known as eudaimonic wellbeing, which refers to the actualization of individuals’ potential or true value as well as the evaluation of one’s circumstance and functioning in the society (Ryff and Keyes, 1995; Keyes, 1998). There are three items in emotional wellbeing (1–3), which are divided into 2 dimensions, namely, positive affect and avowed quality of life. There are five items in social wellbeing (4–8), which are divided into 5 dimensions, namely, social acceptance, social actualization, social contribution, social coherence, and social integration. There are six items in psychological wellbeing (9–14), which are divided into 6 dimensions, namely, self-acceptance, personal growth, purpose in life, environmental mastery, autonomy, and positive relations with others. Participants were asked to evaluate the number of times they felt the problems mentioned in the past 1 month and to respond with “1” representing “never” and “6” representing “every day.” The scores of each subscale for each participant were calculated by summing the scores of the items. The total scores are positively associated with the level of wellbeing. The Chinese version has high validity and reliability and could effectively measure individual wellbeing (Yin and He, 2012).

### Magnetic Resonance Imaging

Magnetic resonance imaging images were collected using the 3.0 T GE scanner Discovery MR750 at the Institute of Psychology, Chinese Academy of Sciences. All the participants completed a T1-weighted structural MRI scan (eyes closed) with a 3D-FSPGR sequence [repetition time (TR) = 6.652 ms; echo time (TE) = 2.928 ms; flip angle (FA) = 12°; matrix = 256 × 256; slice thickness = 1 mm; field of view = 224 mm × 224 mm] and an 8-min resting-state fMRI scan (eyes open with a fixation cross) using a gradient echo EPI sequence [TR = 2,000 ms; TE = 30 ms; FA = 90°; number of slices = 33 (interleaved); slice thickness = 3.5 mm; gap = 0.7 mm; and matrix = 64 × 64].

### Data Preprocessing

Magnetic resonance imaging images were preprocessed using the Connectome Computation System (CCS)^1^ developed by our laboratory (Xu et al., 2015), which integrated three common neuroimaging toolboxes including AFNI (Cox, 2012), FSL (Jenkinson et al., 2012), and FreeSurfer (Fischl, 2012), as well as in-house MATLAB scripts. The CCS pipelines were employed to preprocess all individual images, including both structural and functional images, as well as quality control (Zuo et al., 2013; Xu et al., 2015). The structural processing pipelines mainly included (1) intensity inhomogeneity correction, (2) brain extraction, (3) tissue segmentation, (4) white and pial surface generation, and (5) deformation estimation between the resulting spherical mesh and a common spherical coordinate system. The functional processing pipelines were consistent with our previous publications (Jiang et al., 2015; Zhang et al., 2017) and were mainly included (1) excluding the first 5 volumes from each scan; (2) removing and interpolating time series spikes; (3) correcting slice timing and head motion; (4) extracting functional brain; (5) normalizing 4D global mean of image intensity; and (6) co-registration between functional and anatomical images by employing a boundary-based registration (BBR) algorithm.

### Quality Control Procedure

Following the preprocessed individual MRI images, the CCS also provided a quality control procedure (QCP) for both functional and structural images. The QCP includes the following steps (Zuo et al., 2013): (1) brain extraction or skull stripping, (2) brain tissue segmentation, (3) pial and white surface reconstruction, (4) BBR-based functional image registration, and (5) head motion during r-FMRI. The pipeline also computed the mean frame-wise displacement (meanFD; Power et al., 2012) and the minimal cost of the BBR co-registration (mcBBR) for the subsequent statistical tests as covariates. All participants with bad brain extraction, tissue segmentation, and bad surface construction will be excluded from the subsequent analysis. One participant did not complete MRI scanning, and one participant did not pass the mental health interview. Five participants were excluded because their mcBBR was greater than 0.65. Therefore, we had 60 participants (31 males and 29 females, aged from 19.5 to 64.3 years with an average of 37.8 years, educated from 8 to 22 years with an average of 15.5 years) for final group analysis. The detailed participant information is shown in Table 1.

**TABLE 1:** Participant information of all the 60 subjects including basic demographics, brain characteristics, and wellbeing scores.

Measurements	Average ± SD (range)
Age (Years)	37.8 ± 13.1 (19.5–64.3)
Sex (M/F)	31/29
Education (Years)	15.5 ± 3.1 (8∼22)
meanFD (mm)	0.11 ± 0.05 (0.05∼0.29)
mcBBR	0.57 ± 0.04 (0.48∼0.64)
meanReHo	0.48 ± 0.05 (0.38∼0.64)
meanFC (1)	0.09 ± 0.07 (−0.05∼0.29)
meanFC (2)	0.09 ± 0.07 (−0.04∼0.29)
Psychological wellbeing	23.63 ± 5.94 (6∼30)
Emotional wellbeing	11.33 ± 3.28 (0∼15)
Social wellbeing	19.23 ± 5.34 (4∼25)

### Local Short-Range Functional Connectivity: 2dReHo

To examine the local short-range functional homogeneity, we used surface-based functional homogeneity, namely, 2dReHo, which has been documented in the previous study (Zuo and Xing, 2014). Specifically, for a given vertex in the grid (fsaverage5), we identified its nearest neighbors and computed Kendall’s coefficient of concordance of the rfMRI time series of the nearest neighbors, including the vertex itself, as the local short-range connectivity of this vertex. Every vertex on the cortical surface of both hemispheres was computed to produce 2dReHo maps to characterize the local functional connectivity. In addition, all individual 2dReHo maps were further smoothed with a Gaussian kernel with 6-mm full width at half maximum (FWHM) using the fsaverage5. 2dReHo has been demonstrated to have high robustness against temporal and spatial noise and outliers (Zuo et al., 2013).

We employed a general linear model with the covariates of age, sex, education, meanFD, mcBBR, and global mean 2dReHo to evaluate the associations of 2dReHo with wellbeing. Then, we used the false discovery rate (FDR) method to correct the vertex-wise significance values for each contrast of group analysis. Clusters of smaller than 5 vertices were also excluded. The detailed statistical model is shown in the following equation:


(1) $$\begin{array}{c} Y_{2dReHo}=\beta_{1}\times Wellbeing+\beta_{2}\times X_{age}+\beta_{3}\times X_{sex}+\beta_{4}\\ \;\times X_{education}+\beta_{5}\times X_{meanFD}+\beta_{6}\times X_{mcBBR}+\beta_{7}\\ \;\times X_{meanReHo}+error \end{array}$$

### Remote Long-Range Functional Connectivity: Seed-Based Correlation

Because of the limitations of the only characterization of the local functional connectivity of 2dReHo, to compensate for the lack of this metric, we further developed a local-to-remote cortical connectivity method integrating 2dReHo and the SFC method (Jiang et al., 2015). Each cluster showing effects on 2dReHo was used as a seed region for subsequent functional connectivity analysis. On the basis of individual preprocessed rfMRI time series that were spatially smoothed with 6 mm FWHM, the mean time sequence was computed across all vertices in the cluster, and then we calculated its correlations with the time series of all the other vertices using the fsaverage5. All individual correlation maps were further spatially smoothed with 6 mm FWHM. Similar to 2dReHo, we also used a general linear model with the covariates of sex, age, education, meanFD, mcBBR, and global mean SFC to evaluate the associations of wellbeing with remote cortical connectivity. The same FDR method was used to correct the vertex-wise significance values for each contrast of group analysis. Clusters of smaller than 5 vertices were also excluded.

## Results

### Well-Being Was Associated With Local Cortical Connectivity 2dReHo

**FIGURE 1:**
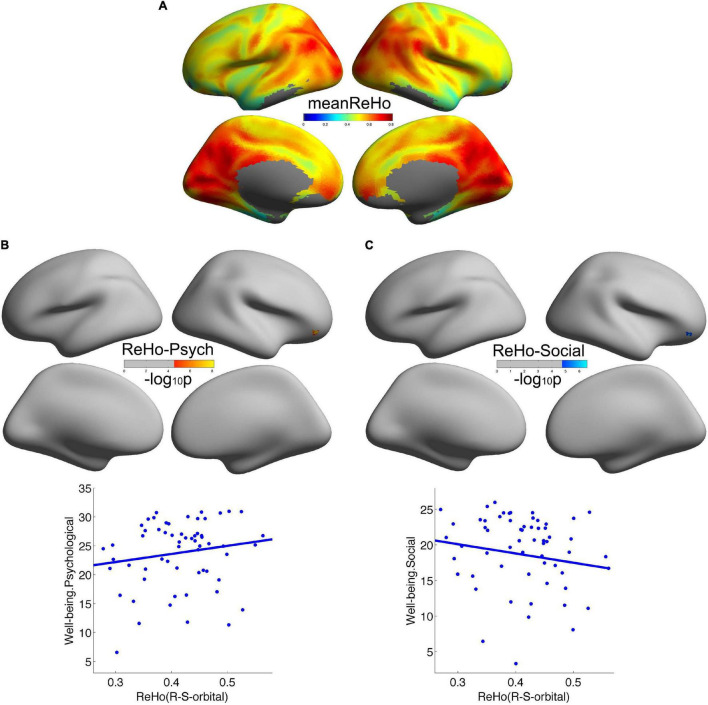
Local cortical connectivity in healthy participants and its associations with wellbeing. **(A)** Showed the average local cortical connectivity 2dReHo across all the participants. 2dReHo in the right orbitofrontal sulcus positively correlated with psychological wellbeing **(B)** but negatively correlated with social wellbeing **(C)**.

Consistent with our previous studies (Jiang et al., 2014), the posteromedial cortex and the parietal cortex exhibited high local functional connectivity ReHo (Figure 1A). In addition, in Figures 1B,C, we observed that local functional connectivity (2dReHo) in the right orbitofrontal sulcus positively correlated with psychological wellbeing but negatively correlated with social wellbeing. No other significant correlations were observed. For a more intuitive illustration of the associations of wellbeing with local functional connectivity, we also depicted scatters of the residues of both wellbeing and 2dReHo in Figure 1. One note was that the vertices with significant correlations of psychological wellbeing were not the same as the vertices with significant correlations of social wellbeing, although they were both within the right orbitofrontal sulcus. Also, we only reported the clusters of more than 5 vertices, and the detailed information of the brain regions with significant correlations between cortical connectivity and wellbeing is illustrated in Table 2.

**TABLE 2:** Brain regions with significant associations of wellbeing with local-to-remote cortical connectivity.

		Brain regions	Vertex number	Size (mm^2^)	−log_10_p	TalX, TalY, TalZ	Corr. r	Effect size	Wellbeing
Local		R_S_orbital	8	110.87	8.178	39.2, 39.0, −11.1	0.1656	0.062	Psychological
		R_S_orbital	6	86.90	−6.555	39.2, 39.0, −11.1	−0.1701	0.280	Social
Remote	Seed of ReHo-psych	L_S_postcentral	10	90.64	5.466	−24.1, −44.6, 56.6	0.3626	0.151	Social
		R_S_orbital	5	72.45	7.191	39.2, 39.0, −11.1	0.2792	0.138	Psychological
	Seed of ReHo-social	L_S_orbital	9	97.44	5.534	−39.2, 28.7, −12.1	0.2501	0.074	Psychological
		R_S_orbital	6	85.04	7.432	39.2, 39.0, −11.1	0.1591	0.128	Psychological

*L, left; R, right; S, sulcus.*

### Well-Being Was Associated With Remote Cortical Connectivity

**FIGURE 2:**
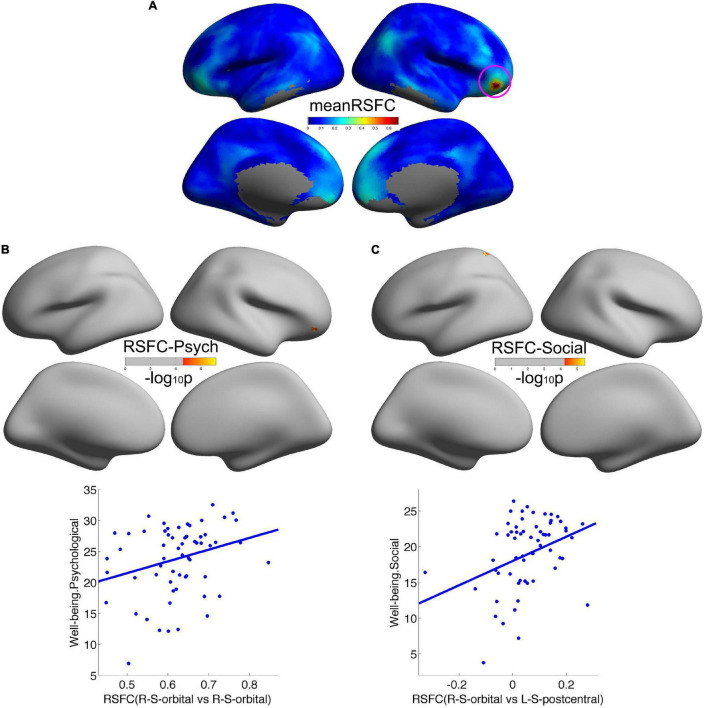
Remote cortical connectivity in healthy participants and its associations with wellbeing. **(A)** Illustrated the average resting-state functional connectivity (RSFC) across all the participants taking the psychological wellbeing-related right orbitofrontal sulcus as the seed region. Remote cortical connectivity within the right orbitofrontal cortex was positively correlated with psychological wellbeing **(B)**, as well as remote cortical connectivity between the right orbitofrontal sulcus and the left postcentral sulcus was positively correlated with social wellbeing **(C)**.

**FIGURE 3:**
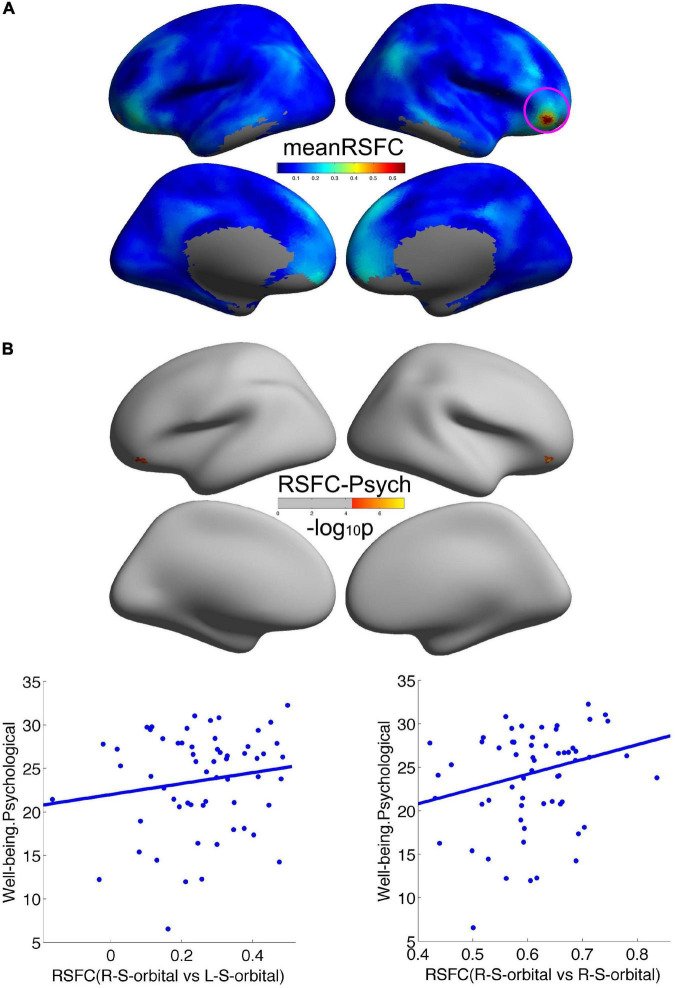
Remote cortical connectivity and its associations with wellbeing. **(A)** Illustrated the average RSFC across all the participants taking the social wellbeing-related right orbitofrontal sulcus as the seed region. The remote cortical connectivity within the right orbitofrontal sulcus and the remote connectivity of contralateral orbitofrontal sulcus were both positively correlated with psychological wellbeing **(B)**.

We employed the right orbitofrontal sulcus showing the effect on psychological wellbeing as seed region, and the seed-based functional connectivity analysis revealed that a significant increment of short-range connectivity with psychological wellbeing was spatially extended to long-range connectivity within the right OFC: within the right OFC, both local and remote cortical connectivity increased with psychological wellbeing. We also observed that the remote cortical connectivity between the right orbitofrontal sulcus and the left postcentral sulcus positively predicted social wellbeing (Figure 2). Additionally, we employed the cluster showing effect on social wellbeing as seed region, and the local-to-remote cortical connectivity results demonstrated that the remote cortical connectivity within the right orbitofrontal sulcus and the remote connectivity of contralateral orbitofrontal sulcus were both positively correlated with psychological wellbeing (Figure 3). We only reported the clusters of more than 5 vertices, and the detailed information of the brain regions with significant correlations between cortical connectivity and wellbeing is illustrated in Table 2.

## Discussion

In this study, we used local-to-remote cortical connectivity to explore whether wellbeing was associated with local-to-remote cortical connectivity. Our analysis revealed that local cortical connectivity (2dReHo) in the right orbitofrontal sulcus not only positively predicted individual differences in psychological wellbeing but also negatively predicted differences in social wellbeing. More importantly, we demonstrated that the local cortical connectivity indeed expanded to remote cortical connectivity: the remote connectivity within the right orbitofrontal sulcus and the remote connectivity of the contralateral orbitofrontal sulcus positively predicted psychological wellbeing, as well as the remote connectivity between the right orbitofrontal sulcus and the left precentral sulcus positively predicted social wellbeing. Taking the significance of anatomical distance in human brain functional architecture into consideration, this study may shed new light on the neural mechanisms underlying wellbeing.

For local cortical connectivity, we discovered that 2dReHo in the right orbitofrontal sulcus was positively correlated with psychological wellbeing but negatively correlated with social wellbeing, suggesting that this region probably contributed to wellbeing-related experience in the brain. On the one hand, the OFC locates the ventral region of the prefrontal cortex, and neuroimaging studies have demonstrated that it played an important role in the regulation and processing of emotion (Bechara et al., 2000; Ochsner et al., 2002, 2004; Kringelbach and Rolls, 2004), encoding rewards of hedonic or painful experience (O’Doherty et al., 2001; Kringelbach, 2005), as well as dispositional mindfulness (Kong et al., 2015b), which were crucial to the integration of appropriate information, including emotions and behaviors to promote individual’s psychological and physical health. Meanwhile, compared with healthy participants, decreased 2dReHo was found in patients with various diseases such as depression (Yao et al., 2009) and migraine (Yu et al., 2012) in the right OFC, which also demonstrated the significance of spontaneous activities of the OFC in individual psychological health. Therefore, healthy participants with higher 2dReHo in the orbitofrontal sulcus have a higher level of psychological wellbeing. On the other hand, the negative relationship between social wellbeing and local cortical connectivity 2dReHo was consistent with prior studies. For example, by using the voxel-based morphometry (VBM) method, researchers found that the regional gray matter volume (rGMV) in the OFC was negatively associated with social wellbeing (Kong et al., 2019). These results consistently verified that the OFC might be a complex site for social wellbeing. Furthermore, the OFC has also been proved to play an important role in social cognition and interaction (Adolphs, 2001; Amodio and Frith, 2006), social cooperation (Rilling et al., 2002), theory of mind and empathy (Sabbagh, 2004; Vollm et al., 2006), and social decision-making such as trust, reciprocity, altruism, and social norm conformity (Sanfey, 2007; Rilling et al., 2008; Rilling and Sanfey, 2011). All of these social-cognitive processes are prerequisites for an individual’s social functioning, and the negative correlation between 2dReHo and social wellbeing in the orbitofrontal sulcus might imply a reorganization mechanism for neuroplasticity and cortical remapping or disruption of inhibitory mechanisms (Xu et al., 2014; Zhou et al., 2014). The negative correlation between the OFC and social wellbeing may also be due to synaptic pruning of redundant neurons during development, which contributed to more efficient information processing in the human brain (Kanai and Rees, 2011).

Based on the above local cortical connectivity associations with wellbeing, our remote cortical connectivity findings confirmed that the local short-range functional connectivity indeed diffused remote long-range functional connectivity in individual wellbeing. Previous studies on the cortical functional connectivity have revealed that anatomical distance played an important role in designing the functional architecture of the human brain (Ercsey-Ravasz et al., 2013; Samu et al., 2014). Also, local short connections and remote long connections were, respectively, related to within-module and intermodular connections in different cortical hierarchies (Alexander-Bloch et al., 2013; Sporns, 2013). Local-to-remote cortical connectivity has also been demonstrated to be correlated with various diseases such as schizophrenia (Sigurdsson et al., 2010; Jiang et al., 2015), chronic insomnia disorder (Zhou et al., 2020), renal disease (Chen et al., 2020), Parkinson’s disease (Harrington et al., 2020), and Alzheimer’s disease (Dai et al., 2015). Our findings demonstrated, for the first time, that wellbeing was associated with the local-to-remote cortical connectivity of the human brain. We found that the remote long-range connectivity between the right OFC and the left postcentral sulcus was positively correlated with social wellbeing. The postcentral sulcus locates the primary somatosensory cortex, which played an important role in touch, temperature, pain, and proprioception (Kreisman and Zimmerman, 1973; Ploner et al., 2000). Therefore, the cultivation of social wellbeing might need the integration and processing of sensory information and social cognition that led to the remote functional connectivity of these two brain regions. The local-to-remote method to investigate the short- and long-range functional connectivity of wellbeing, which has not been employed in previous wellbeing-related studies, could extend and enrich our knowledge of the neural mechanisms of wellbeing and provide novel insights in future neuroimaging studies.

Among the multiple remote long-range functional connectivity associations, the most remarkable one was that interhemispheric functional connectivity between the contralateral orbitofrontal sulcus positively correlated with psychological wellbeing. This kind of connectivity between contralateral brain regions, called functional homotopy, has been noticed and deeply investigated in previous studies: as a fundamental characteristic of the intrinsic human brain architecture (Stark et al., 2008), functional homotopy reflects high degree of synchrony in spontaneous activity between geometrically corresponding (i.e., homotopic) regions in each hemisphere (Zuo et al., 2010). Also, impairments of interhemispheric homotopic connectivity have been reported in various neuropsychiatric disorders such as obsessive-compulsive disorder (OCD; Jia et al., 2020), cervical dystonia (Jiang et al., 2019), depressive disorder (Guo et al., 2018), somatization disorder (Su et al., 2016), and autism spectrum disorder (Li et al., 2019). All these validated that the homotopic connectivity might be one of the basic organizational principles of neural underpinning. Interestingly, in this study, we found remote functional connectivity of the contralateral orbitofrontal sulcus with psychological wellbeing, which was consistent with other studies that also demonstrated significant homotopic connectivity changes in the OFC (Jiang et al., 2020). In summary, the strong connection between homotopic brain regions within the OFC in healthy participants might contribute to the integration of emotional and cognitive information to promote individual wellbeing.

Using the local-to-remote cortical connectivity method, we concluded that wellbeing was associated with local-to-remote cortical connectivity, especially when we identified the OFC as a key region for wellbeing characterization. However, our study still had several limitations: first, we focused only on the cortical connectivity of the human brain, and in future, we might take subcortical structures into account to investigate whole-brain connectivity. Second, our sample size was not large, and we need to recruit more participants to get a more convincing result and a better statistical power. Finally, the current wellbeing measurement depends on participants’ self-report. Subjective wellbeing, which belongs to a category of psychology, is not a good guideline for understanding how the human brain works or how subjective wellbeing works, and it is necessary to employ more objective cognitive or emotional tests to get a deeper understanding of wellbeing itself as well as the associations of wellbeing with the human brain.

## Conclusion

In this study, we used the local-to-remote cortical connectivity method to explore whether wellbeing was associated with local to remote cortical connectivity. Our results showed that the local connectivity in the right orbitofrontal sulcus was significantly positively correlated with psychological wellbeing but negatively correlated with social wellbeing; the remote connectivity within the right OFC and interhemispheric connectivity of the orbitofrontal sulcus were both positively associated with psychological wellbeing; the remote functional connectivity between the right orbitofrontal sulcus and the left postcentral sulcus was positively associated with social wellbeing. Our results showed that wellbeing was indeed associated with local-to-remote cortical connectivity, and our findings supplied a new perspective of distance-related neural mechanisms of wellbeing.

## Data Availability Statement

The raw data supporting the conclusions of this article will be made available upon request from the corresponding author.

## Ethics Statement

The studies involving human participants were reviewed and approved by the Institutional Review Board of Institute of Psychology, Chinese Academy of Sciences. The patients/participants provided their written informed consent to participate in this study.

## Author Contributions

LJ: conceptualization and methodology. LJ, YL, and CL: formal analysis, investigation, and writing–review and editing. All authors contributed to the article and approved the submitted version.

## Conflict of Interest

The authors declare that the research was conducted in the absence of any commercial or financial relationships that could be construed as a potential conflict of interest.

## Publisher’s Note

All claims expressed in this article are solely those of the authors and do not necessarily represent those of their affiliated organizations, or those of the publisher, the editors and the reviewers. Any product that may be evaluated in this article, or claim that may be made by its manufacturer, is not guaranteed or endorsed by the publisher.

## Footnotes

1
http://github.com/zuoxinian
