# Supplementary figures and images for: Well-Being Is Associated With Local to Remote Cortical Connectivity
Source: Front Behav Neurosci. 2022 Mar 11;16:737121. doi: 10.3389/fnbeh.2022.737121 (PMC8967134; doi:10.3389/fnbeh.2022.737121)

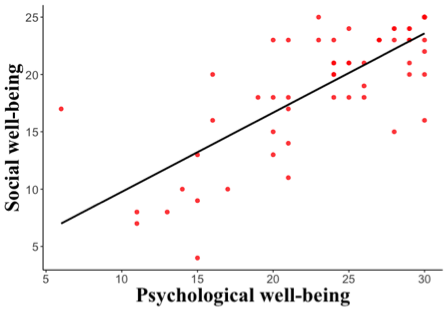

Supplement: Supplementary file 1 [file Image_1.tiff]
