# Supplementary material for: Well-Being Is Associated With Local to Remote Cortical Connectivity
Source: Front Behav Neurosci. 2022 Mar 11;16:737121. doi: 10.3389/fnbeh.2022.737121 (PMC8967134; doi:10.3389/fnbeh.2022.737121)
Supplement: Supplementary file 2 [file Table_1.docx]

**Table S1.** The Pearson correlation coefficients among different dimensions of well-being.

| Variables | Total scores of well-being | Emotional well-being | Psychological well-being |
| --- | --- | --- | --- |
| Total scores of well-being | 1 |  |  |
| Emotional well-being | 0.857** | 1 |  |
| Psychological well-being | 0.930** | 0.713** | 1 |
| Social well-being | 0.921** | 0.718** | 0.760** |

*Note. Well-being, total scores of well-being*
